# Comparing Obstetrical Outcomes Between Attention Deficit Hyperactivity Disorder and Attention Deficit Disorder: A Population-Based Studys

**DOI:** 10.3390/jcm14124142

**Published:** 2025-06-11

**Authors:** Uri Amikam, Ahmad Badeghiesh, Haitham Baghlaf, Richard Brown, Michael H. Dahan

**Affiliations:** 1 Department of Obstetrics and Gynecology, McGill University, Montréal, QC H3A 0G4, Canada; richard.brown@mcgill.ca (R.B.); thedoctormichaeldahan@gmail.com (M.H.D.); 2 The Faculty of Medicine, Tel Aviv University, Tel Aviv 6997801, Israel; 3 Department of Obstetrics and Gynecology, King Abdulaziz University, Rabigh Branch, Rabigh 25732, Saudi Arabia; badeghiesh@gmail.com; 4 Department of Obstetrics and Gynecology, University of Tabuk, Tabuk 47512, Saudi Arabia; baghlafh@gmail.com

**Keywords:** attention deficit hyperactivity disorder, attention deficit disorder without hyperactivity, neurodevelopmental disorder, preterm delivery, hypertensive disorders of pregnancy, small-for-gestational-age

## Abstract

**Objectives**: Attention deficit hyperactivity disorder (ADHD) is among the most common neurodevelopmental disorders affecting women of reproductive age. Previous data on this condition did not study its different symptom clusters separately. Our aim was to compare perinatal outcomes between women with hyperactivity cluster (ADHD) and those with the inattentive cluster (attention deficit disorder (ADD)). **Methods**: A retrospective population-based study utilizing data from the Healthcare Cost and Utilization Project–Nationwide Inpatient Sample (HCUP-NIS). All deliveries or maternal deaths from 2004 to 2014 were available for analysis, and perinatal outcomes were compared between participants with an ADD diagnosis and those with an ADHD diagnosis. A multivariate logistic regression was used to control for confounders. **Results**: During the study period, there were 9,096,788 deliveries. Of them, 7103 had an ADHD diagnosis, and 2928 had an ADD diagnosis. Women with ADHD, compared to those with ADD, were more likely to be younger than 25 years of age; to be Black; to be from a lower income quartile; to smoke tobacco during pregnancy; and to use illicit drugs (*p* < 0.001 for all). Using multivariate logistic regression, women with ADHD, compared to those with ADD, had a higher rate of hypertensive disorders of pregnancy (HDPs) (aOR 1.19, 95% CI 1.03–1.37, *p* = 0.02), preterm delivery (aOR 1.19, 95% CI 1.01–1.39, *p* = 0.038), maternal infection (aOR 1.39, 95% CI 1.04–1.85, *p* = 0.024), and small-for-gestational-age (SGA) neonates (aOR 1.33, 95% CI 1.04–1.69, *p* = 0.022). **Conclusions**: Women with an ADHD diagnosis, compared to those with ADD, had a higher incidence of various maternal and neonatal complications, including HDPs, preterm delivery, and SGA neonates.

## 1. Introduction

Attention deficit hyperactivity disorder (ADHD) is a common neurodevelopmental disorder affecting about 5% of children worldwide and persisting into adulthood with a prevalence of about 3% globally [1,2,3]. This estimate encompasses individuals diagnosed with ADHD in childhood whose symptoms continue into adulthood, as well as cases where ADHD is diagnosed in adulthood.

The most updated diagnosis of ADHD was published in 2013 in the Psychiatric Association’s Diagnostic and Statistical Manual, Fifth edition (DSM-5) [4]. It relies on the identification of pervasive, developmentally excessive, and impairing levels of impulsivity, inattention, and overactivity. ADHD is primarily categorized into three subtypes: primarily inattentive, primarily hyperactive–impulsive, or a combination of both [5]. These three subtypes reflect different symptomatic profiles. The Predominantly Inattentive Presentation is distinguished by pervasive difficulties with attention, organizational skills, and sustained focus; the Predominantly Hyperactive–Impulsive Presentation is primarily marked by hyperactivity, impulsivity, and difficulty with inhibitory control; and the Combined Presentation exhibits a constellation of symptoms from both inattention and hyperactivity–impulsivity domains [4,6,7]. Recognizing these subtypes is essential for an accurate diagnosis and individualized treatment planning.

The term attention deficit disorder (ADD) was initially introduced in the third edition of the DSM (DSM-3), where two subtypes of ADD were identified: ADD with hyperactivity and without. Subsequently, a revised version of the DSM (DSM-3-R) was published, and the term ADHD was initially introduced with the removal of ADD [8]. The removal of ADD from the DSM resulted in criticism [9], and despite being removed, ADD is still utilized in practice and has a specific International Classification of Disease, Ninth Revision, Clinical Modification (ICD-9-CM) code, which is still being used. The ADD diagnosis is currently considered as ADHD of the Predominantly Inattentive Presentation.

There has been a notable rise in the prevalence of ADHD among both children and adults in recent decades within the United States [10,11], with some studies suggesting a more rapid increase among women compared to men [12,13]. These increases might signify either a genuine escalation in the actual prevalence of ADHD over time or improved rates of diagnosis, possibly attributed to a better recognition of underlying ADHD [1,10]. Irrespective of the cause behind the observed increase, the anticipated consequence is a rise in the incidence of pregnant women diagnosed with ADHD.

ADHD can significantly impact a person’s life from childhood into adulthood, affecting both their personal growth and social interactions. In childhood, ADHD typically manifests as challenges with attention, hyperactivity, and impulsivity, which can affect academic achievement [6]. Children with ADHD may struggle to concentrate on tasks, follow instructions, or complete assignments, often resulting in lower grades and increased frustration [14]. The social consequences are also significant; these children may struggle to form and sustain friendships due to impulsive behaviors and difficulties reading social cues [15]. This can lead to feelings of isolation or diminished self-esteem, which can negatively impact their overall emotional well-being.

As individuals with ADHD transition into adulthood, the condition can continue to influence many areas of life, including work, relationships, and daily functioning. Psychosocial aspects are significantly impacted, as adults with ADHD may experience difficulties with time management, organization, and task prioritization, which can obstruct career advancements and lead to job instability [16]. Deficits in executive functions, such as planning, impulse control, and emotional regulation, also present challenges in personal relationships and social interactions [6,17]. Adults may struggle with restlessness and often find it challenging to remain engaged in conversations or social activities. This can perpetuate a cycle of stress and anxiety, affecting mental health [18].

While previous studies explored pregnancy complications in women with an ADHD diagnosis, including increased risks for preeclampsia, gestational diabetes mellitus (GDM), and a cesarean delivery (CD) [2,19], the data comparing the pregnancy outcomes in women with ADD compared to women with ADHD are scarce.

Jones et al. examined how different symptom clusters of ADHD predict prenatal behavior and found that hyperactivity was positively associated with caffeine use, smoking, and physical strain and negatively associated with prenatal vitamin usage [20]. Whether the hyperactivity component has different adverse pregnancy outcomes compared to ADD is a question that, to date, remains unanswered.

Considering the paucity of data in the literature, our objective was to evaluate maternal and neonatal outcomes in women with ADHD compared to those with an ADD diagnosis, utilizing an extensive nationwide database from the United States.

## 2. Materials and Methods

### 2.1. Study Design

We conducted a retrospective population-based cohort study, using data from the Healthcare Cost and Utilization Project Nationwide Inpatient Sample (HCUP-NIS). The HCUP-NIS constitutes the most extensive inpatient sample database in the United States. It encompasses submissions of hospital inpatient stays from 1000 hospitals, which collectively represent approximately 20% of hospital admissions and geographically account for over 96% of the American population.

According to articles 2.2 and 2.4 of the Tri-Council Policy Statement (2010) [21], an institutional review board approval was not required, as this study exclusively used publicly accessible, anonymized data.

The requirement for informed consent was waived because of the retrospective design of this study.

### 2.2. Study Participants and Data Collection

The cohort comprised pregnant women delivering between the years 2004 and 2014, and it was restricted to admissions resulting in the event of delivery or maternal death, ensuring that multiple admissions in the same pregnancy were excluded from the study.

We included in the study women with either an ADD or ADHD diagnosis, and it was divided into two groups based on each of these diagnoses. Women with ADD and ADHD were identified using the ICD-9-CM diagnoses, with the codes 314.00 for ADHD and 314.01 for ADD (predominantly inattentive type).

The data collected included demographic and obstetric parameters, specifically regarding the labor process, as well as immediate maternal and neonatal outcomes up to the point of the patient’s admission and subsequent hospital discharge. Baseline maternal clinical characteristics included patient age, race, income divided into four quartiles, type of insurance, obesity (defined as a body mass index (BMI) ≥ 30 kg/m^2^), previous CD, multiple gestation, tobacco smoking during pregnancy, illicit drug use, pre-existing hypertension, pre-existing diabetes, pre-existing thyroid disease, human immunodeficiency virus status, and use of in vitro fertilization to conceive the pregnancy.

Parameters regarding labor and delivery encompassed hypertensive disorders of pregnancy (HDPs), including any of the following diagnoses: gestational hypertension, preeclampsia, eclampsia, and preeclampsia and eclampsia superimposed on hypertension; GDM; preterm delivery (PTD) (<37 weeks); preterm premature rupture of membranes; CD; placenta previa; postpartum hemorrhage (PPH); disseminated intravascular coagulation; wound complications; maternal infection; venous thromboembolism; and deep vein thrombosis. The neonatal outcomes that were included in the study were small-for-gestational-age (SGA) neonates; intra-uterine fetal death (IUFD); and congenital anomalies.

### 2.3. Statistical Analysis

Initially, chi-square tests were performed to compare the baseline characteristics of women with an ADHD diagnosis and those with an ADD diagnosis. The following continuous parameters were converted into categorical parameters: age, BMI, and income. Therefore, they were not checked for normal distribution. Subsequently, logistic regression analyses were conducted to compare delivery and neonatal outcomes between the two study groups by estimating odds ratios (ORs) and 95% confidence intervals (CIs). The regression models were adjusted to account for the potential confounding effects of maternal demographics, pre-existing clinical characteristics, and concurrently occurring conditions that reached statistical significance (*p* < 0.05) on the chi-squared tests in the initial analysis. SPSS 25.0 (IBM SPSS Statistics 25, Armonk, NY, USA: IBM Corp) was used to perform all analyses.

The HCUP data is publicly available. Per HCUP guidelines, when there were fewer than 11 cases of a complication, it was represented as “<11” to maintain patient anonymity. However, if there were zero cases, anonymity was not a concern.

### 2.4. Data Synthesis

This study’s findings are presented in three tables. Table 1 outlines the baseline maternal characteristics. Pregnancy and delivery outcomes are presented in Table 2, while Table 3 outlines the neonatal outcomes.

## 3. Results

In total, 9,096,788 deliveries were recorded in the database from 2004 to 2014 inclusively. Of these, 7103 women had an ADHD diagnosis, and 2928 had an ADD diagnosis. Table 1 depicts the baseline and demographic characteristics of women with ADHD and ADD diagnoses. Women with ADHD, compared to those with ADD, were younger; more likely to be Black; more likely to be in the lower income quartile; more likely to have Medicare or Medicaid health insurance; and more likely to consume illicit drugs or smoke tobacco during pregnancy (*p* < 0.001 for all). Other maternal characteristics, including the prevalence of obesity, chronic hypertension, pregestational DM, thyroid disorders, and previous CD, were comparable between the two groups.

**Table 1:** Baseline maternal characteristics.

Characteristics	Attention Deficit Disorder with Hyperactivity N = 7103	Attention Deficit Disorder Without Hyperactivity N = 2928	*p*-Value
Age (years)			<0.001
<25	4371 (61.5%)	1456 (49.7%)	
25–34	2201 (31%)	1156 (39.5%)	
≥35	531 (7.5%)	316 (10.8%)	
Race ^1^			<0.001
White	5177 (72.9%)	2331 (79.6%)	
Black	1081 (15.2%)	317 (10.8%)	
Hispanic	561 (7.9%)	172 (5.9%)	
Asian and Pacific	82 (1.2%)	23 (0.8%)	
Native American	39 (0.5%)	17 (0.6%)	
Other	144 (2%)	54 (1.8%)	
Income quartiles			<0.001
Less than 39,000	1898 (26.7%)	559 (19.1%)	
USD 39,000–47,999	2242 (31.6%)	893 (30.5%)	
USD 48,000–62,999	1764 (24.8%)	850 (29%)	
USD 63,000 or more	1199 (16.9%)	626 (21.4%)	
Plan type			<0.001
Medicare	271 (3.8%)	77 (2.6%)	
Medicaid	3998 (56.3%)	1215 (41.5%)	
Private including HMO	2483 (35%)	1478 (50.5%)	
self-pay	117 (1.6%)	40 (1.4%)	
No charge	<11	<11	
Other	232 (3.3%)	116 (4%)	
Obesity (BMI ≥ 30 Kg/m^2^)	645 (9.1%)	246 (8.4%)	0.277
Previous CD	881 (12.4%)	400 (13.7%)	0.086
Tobacco Smoking during pregnancy	1830 (25.8%)	572 (19.5%)	<0.001
Chronic hypertension	177 (2.5%)	80 (2.7%)	0.489
Pregestational DM	114 (1.6%)	42 (1.4%)	0.53
Illicit drug use	754 (10.6%)	200 (6.8%)	<0.001
Multiple gestation	107 (1.5%)	48 (1.6%)	0.624
Thyroid disease	308 (4.3%)	148 (5.1%)	0.116
HIV	<11	<11	0.854
IVF	<11	<11	0.595

Abbreviations and definitions: HMO—Health Maintenance Organization; BMI—body mass index; CD—cesarean delivery; DM—diabetes mellitus; HIV—human immunodeficiency virus; and IVF—in vitro fertilization. ^1^ Due to the rounding of numbers, the percentages do not add up to 100%.

Per the convention of the HCUP database, when N < 11, the absolute cell number of subjects was not provided to protect patient anonymity.

The comparison between ADHD and ADD regarding pregnancy and delivery outcomes is presented in Table 2. The pregnancy and delivery outcomes are presented both before and after adjusting for potential confounders, including maternal age, race, income quartiles, insurance plan types, illicit drug use during pregnancy, and tobacco smoking, with the addition of HDPs for the analysis of delivery and neonatal outcomes. Women with an ADHD diagnosis, compared to those with ADD, had a higher rate of HDPs (adjusted OR (aOR) 1.19, 95% CI 1.03–1.37, *p* = 0.02); PTD (aOR 1.19, 95% CI 1.01–1.39, *p* = 0.038); and maternal infection (aOR 1.39, 95% CI 1.04–1.85, *p* = 0.024). Other pregnancy and delivery outcomes examined, such as GDM, CD, PPH, hysterectomy, venous thromboembolism, and the need for blood product transfusion, were similar between the groups.

**Table 2:** Pregnancy and delivery outcomes.

Outcomes	Attention Deficit Disorder with Hyperactivity (%)	Attention Deficit Without Hyperactivity Disorder (%)	Crude OR (95% CI)	Adjusted OR (95% CI)	Adjusted *p*-Value
**Pregnancy outcomes ^a^**					
HDPs	797 (11.2%)	295 (10.1%)	1.13 (0.98–1.3)	1.19 (1.03–1.37)	0.02
GDM	351 (4.9%)	146 (5%)	0.99 (0.81–1.21)	1.12 (0.91–1.38)	0.269
Placenta previa	44 (0.6%)	17 (0.6%)	1.07 (0.61–1.87)	1.05 (0.59–1.86)	0.869
**Delivery outcomes ^b^**					
PPROM	110 (1.5%)	40 (1.4%)	1.14 (0.79–1.64)	1.07 (0.74–1.56)	0.709
Preterm delivery	675 (9.5%)	227 (7.8%)	1.25 (1.07–1.46)	1.19 (1.01–1.39)	0.038
Abruptio placenta	112 (1.6%)	30 (1%)	1.55 (1.03–2.32)	1.4 (0.93–2.12)	0.109
Chorioamnionitis	181 (2.5%)	55 (1.9%)	1.37 (1.01–1.85)	1.37 (1–1.86)	0.05
Operative vaginal delivery	355 (5%)	161 (5.5%)	0.91 (0.75–1.1)	0.9 (0.74–1.1)	0.314
CD	2381 (33.5%)	1030 (35.2%)	0.93 (0.85–1.02)	0.96 (0.88–1.06)	0.433
SVD	4367 (61.5%)	1737 (59.3%)	1.09 (1.01–1.2)	1.06 (0.97–1.16)	0.227
Hysterectomy	<11	<11	0.31 (0.07–1.39)	0.41 (0.09–1.92)	0.257
PPH	272 (3.8%)	93 (3.2%)	1.21 (0.96–1.54)	1.22 (0.95–1.55)	0.119
Wound complications	40 (0.6%)	19 (0.6%)	0.87 (0.5–1.5)	0.91 (0.52–1.6)	0.752
Maternal Death	<11	0 (0%)	N/A	N/A	N/A
Transfusion	92 (1.3%)	26 (0.9%)	1.47 (0.95–2.28)	1.33 (0.85–2.09)	0.205
**Others**					
Maternal infection	221 (3.1%)	65 (2.2%)	1.41 (1.07–1.87)	1.39 (1.04–1.85)	0.024
DVT	<11	<11	0.82 (0.15–4.5)	1.08 (0.19–6.02)	0.932
PE	<11	0 (0%)	N/A	N/A	N/A
VTE	<11	<11	1.44 (0.3–6.95)	1.7 (0.35–8.39)	0.513
DIC	13 (0.2%)	<11	0.59 (0.25–1.39)	0.53 (0.22–1.26)	0.148

^a^ Pregnancy Outcomes: adjusted for age, race, plan type, income quartiles, illicit drug use, and tobacco smoking in pregnancy. ^b^ Delivery Outcomes: adjusted for age, race, plan type, income quartiles, illicit drug use and tobacco smoking in pregnancy, and hypertensive disorders of pregnancy. Abbreviations and definitions: HDPs—hypertensive disorders of pregnancy; GDM—gestational diabetes mellitus; PPROM—preterm premature rupture of membranes; CD—cesarean delivery; SVD—spontaneous vaginal delivery; PPH—postpartum hemorrhage; DVT—deep vein thrombosis; PE—pulmonary embolism; N/A—not applicable; VTE—venous thromboembolism; and DIC—disseminated intravascular coagulation. Per the convention of the HCUP database, when N < 11, the absolute cell number of subjects was not provided to protect patient anonymity (zero subjects could be reported because it would not affect anonymity). In cases with zero occurrences of a particular finding, the OR and CI could not be calculated and were represented as “N/A”.

Table 3 presents neonatal outcomes. Women with an ADHD diagnosis, compared to those with ADD, had a higher rate of SGA neonates (aOR 1.33, 95% CI 1.04–1.69, *p* = 0.022). The rates of congenital anomalies and IUFD were comparable between groups.

**Table 3:** Neonatal outcomes ^a^.

Outcomes	Attention Deficit Disorder with Hyperactivity (%)	Attention Deficit Disorder with Hyperactivity (%)	Crude OR (95% CI)	Adjusted OR (95% CI)	Adjusted *p*-Value
SGA	319 (4.5%)	91 (3.1%)	1.47 (1.16–1.86)	1.33 (1.04–1.69)	0.022
IUFD	29 (0.4%)	12 (0.4%)	0.99 (0.51–1.96)	0.81 (0.41–1.62)	0.554
Congenital Anomalies	108 (1.5%)	41 (1.4%)	1.09 (0.76–1.56)	1.14 (0.79–1.65)	0.483

^a^ Adjusted for age, race, plan type, income quartiles, illicit drug use and tobacco smoking in pregnancy, and hypertensive disorders of pregnancy. Abbreviations and definitions: SGA—small for gestational age; IUFD—intra-uterine fetal death.

## 4. Discussion

Our study evaluates maternal and neonatal outcomes in women with ADHD compared to those with an ADD diagnosis, utilizing an extensive nationwide database from the United States.

Women in the ADHD group were more likely to be younger and of a Black race compared to women with an ADD diagnosis. It was previously described that women with an ADHD diagnosis have a two-fold increased risk of pregnancy under the age of 18 compared to controls, with substance abuse significantly contributing to this finding; albeit, they did not specify the different components of ADHD [22]. Notably, in our cohort, women with an ADHD diagnosis indeed had a higher rate of illicit drug use compared to women with ADD. Previous studies examining the race distribution among ADHD patients found race disparities, with Asian, Black, and Hispanic children significantly less likely to be diagnosed with ADHD compared with White children [23,24]. However, data on the racial distribution among pregnant women with an ADHD diagnosis is sparse [25]. Furthermore, there is currently no available data on the distribution of the different subtypes of ADHD among various races in participants.

We found significantly higher rates of tobacco smoking and illicit drug use during pregnancy in the ADHD group compared to ADD. The rates of tobacco smoking during pregnancy in the study groups were 25.8% and 19.5% in the ADHD and ADD groups, respectively. These rates are significantly higher than the 4.9% rate in the general HCUP pregnant women population without a diagnosis of ADHD or ADD [25]. ADHD symptoms are a known risk factor for tobacco smoking [26,27]. Furthermore, a previous study examining ADHD symptoms in pregnant women, and their potential influence on health behaviors [20], found hyperactivity to be significantly linked with smoking, possibly explaining the significantly higher rate among women with ADHD compared to women diagnosed with ADD. Similarly to tobacco smoking, illicit drug use was also described more frequently among patients with ADHD symptoms [28,29], with a higher incidence among those with hyperactive/impulsive symptoms [30], albeit there is no previous data regarding the effect of different ADHD clusters on the rate of drug use during pregnancy. Our findings emphasize the importance of preconception counseling and screening for harmful prenatal behaviors in these individuals and addressing them accordingly during the prenatal period, with particular attention being paid to those with hyperactivity symptoms.

Women with an ADHD diagnosis had increased rates of HDPs compared to women with ADD, even after adjusting for potential confounders such as a Black race, lower income quartile, and illicit drug use. While previous studies found that the ADHD group as a whole had an increased risk of developing HDPs compared to controls without ADHD [2,25], there are no previous data regarding the effect of the different ADHD clusters on the risk of developing HDPs. There are some possible explanations for this finding. Firstly, an earlier study found an association between hyperactivity/impulsivity symptoms and unplanned pregnancies [31], which is itself a risk factor for HDPs [32]. Secondly, women with hyperactivity symptom clusters were found to be less adherent to prenatal vitamin usage, which has been found to reduce the risk for preeclampsia [33], one of the components of HDPs.

Women in the ADHD group had higher rates of PTD compared to women in the ADD group. While previous studies found an increased risk for PTD among the entire group of women with ADHD [2,34], again its different clusters have not been studied independently. Women with ADHD were found to be at a higher risk for various sexually transmitted infections (STIs), including Gonorrhea, Chlamydia, and Trichomoniasis [35], which are risk factors for PTD [36]. Furthermore, Chen et al. [35] found that patients with ADHD who also had substance use disorders were at the highest risk of acquiring STIs. Women with an ADHD diagnosis in our study had a higher rate of illicit drug use compared to women with ADD. Since we do not have information regarding previous STIs in our database, it will remain unanswered if this is the main cause for the higher PTD rate in the ADHD group. If there were higher rates of STIs in the ADHD group, this could potentially also account for the higher rates of maternal infection observed in this group. Notably, there was a trend towards a higher rate of chorioamnionitis in the ADHD group that did not reach statistical significance (*p* = 0.05). These findings would suggest that an ADHD diagnosis justifies increased surveillance and screening for STIs before and during pregnancy, with the focus being on women with predominant hyperactivity, although both this and the impact of such screening and treatment on the rates of preterm births require further investigation.

Regarding neonatal outcomes, we found higher rates of SGA neonates in women with ADHD compared to women with ADD. While previous studies showed conflicting results regarding SGA in the entire ADHD group [19,25], there is no data describing SGA rates according to the disorder’s different clusters. There are some plausible explanations for our findings. Firstly, a previous study found that hyperactivity symptoms were related to higher rates of unintended pregnancy [31], which is associated with low birthweights [37]. Secondly, although we adjusted for possible confounders associated with SGA, including tobacco smoking and illicit drug use, there could be other possible parameters that are not reported in our database that are related to risky behavior and could potentially affect fetal growth, such as alcohol consumption.

An additional possible explanation for the worse outcomes observed in the ADHD group compared to the ADD group is the differing incidence of psychostimulant use between these groups. A prior study identified an 11-fold increase in the use of ADHD stimulant medications during pregnancy from 2000 to 2021 [38]. To date, there are no studies examining the incidence of medication use for ADHD during pregnancy across different clusters. Evidence suggests that ADHD medications during pregnancy may be linked to placental complications, including preeclampsia and preterm birth [39]. The correlation between the increased risk of adverse pregnancy outcomes observed in the ADHD group and medication use necessitates further exploration in subsequent studies.

### 4.1. Limitations

Our study has several limitations. Firstly, medications are not included in our database, including pharmacotherapy for ADHD and ADD, which is mainly centered around the use of psychostimulants, including methylphenidate or amphetamine derivatives [3]. While this crucial information could influence our findings, as previously discussed, we can presume that its overall impact is likely limited since the same groups of psychostimulants serve as treatments for both the hyperactive/impulsive and inattentive clusters. Consequently, their effects are likely to apply to both groups, likely diminishing their influence on the study outcomes. Secondly, this study’s assumption is that women with an ADD diagnosis had ADHD with predominantly inattentive symptoms. It is possible that some of the women in the ADHD group had an inattentive cluster or a combination of the two clusters, but the code used for their diagnosis was ADHD since ADD was officially removed from the DSM. If this indeed is the case, the inclusion of actual ADD patients in the ADHD group would likely weaken the observed adverse pregnancy outcomes in the ADHD group, further reinforcing the validity of our findings. Conversely, the likelihood of women with predominantly hyperactive symptoms being misdiagnosed as having ADD is lower, as the ADD diagnosis specifically pertains to the inattentive type and does not appear in the latest DSM classification. Lastly, our cohort was restricted until 2014, since later data had differential coding within the HCUP, using ICD-10. Therefore, restricting our analysis to data before 2015 ensured consistency in the coding methodology throughout our cohort.

### 4.2. Strengths

Nonetheless, our study has several strengths. Firstly, the novelty of our study is that, to date, it is the only study examining the pregnancy outcomes among different ADHD clusters. Secondly, it is based on a large cohort spanning a relatively long period, which allows for the detection of differences between groups. Thirdly, given that our data are derived from a population-based cohort, the findings possess a general applicability to various American populations and can additionally provide insights for other societies. Finally, our study allowed for the investigation of a wide spectrum of pregnancy and delivery complications. This depth of analysis equips physicians with detailed information, enabling them to offer more tailored counseling to pregnant women diagnosed with different clusters of ADHD.

### 4.3. Conclusions

In summary, our findings indicate that women diagnosed with the hyperactivity cluster of ADHD face a higher risk of obstetrical complications compared to those diagnosed with ADD. These complications include HDPs, PTD, and maternal infection, along with an increased risk of delivering SGA neonates. These findings underscore the significance of thorough patient counseling, screening for risky behaviors, and multidisciplinary care involving obstetricians, psychiatrists, and allied mental health professionals. Additionally, tailored counseling for each cluster—hyperactivity and inattentive—should be considered.

### 4.4. Implications for Future Research

We believe that future directions in this field should focus on examining obstetric and neonatal outcomes in each cluster independently, rather than viewing ADHD as a unified entity.

## Data Availability

The data that support the findings of this study are available from the corresponding author upon reasonable request.

## Abbreviations

The following abbreviations are used in this manuscript:

ADHD	Attention deficit hyperactivity disorder
ADD	Attention deficit disorder
HCUP-NIS	Healthcare Cost and Utilization Project–Nationwide Inpatient Sample
HDPs	Hypertensive disorders of pregnancy
ICD	International Classification of Disease
GDM	Gestational diabetes mellitus
CD	Cesarean delivery
BMI	Body mass index
PTD	Preterm delivery
PPH	Postpartum hemorrhage
SGA	Small-for-gestational-age
IUFD	Intra-uterine fetal death
OR	Odds ratio
CI	Confidence interval
HMO	Health Maintenance Organization
DM	Diabetes mellitus
HIV	Human immunodeficiency virus
IVF	In vitro fertilization
PPROM	Preterm premature rupture of membranes
SVD	Spontaneous vaginal delivery
DVT	Deep vein thrombosis
PE	Pulmonary embolism
N/A	Not applicable
VTE	Venous thromboembolism
DIC	Disseminated intravascular coagulation
